# Does digital-economy development improve air quality in Border Regions? Empirical evidence from 188 Chinese cities

**DOI:** 10.1371/journal.pone.0348514

**Published:** 2026-05-14

**Authors:** Huifu Li, Mengling Li, Xu Li, Danyang Li

**Affiliations:** Business School, Changsha Social Work College, ‌‌Changsha, ‌‌China; University of Jinan, CHINA

## Abstract

Using panel data from Chinese inter-provincial border cities from 2014 to 2023, this study systematically analyzes the impact of digital-economy development on air quality in border regions. Empirical results show that the digital economy significantly improves air quality in such regions, with this effect being more prominent in non-contiguous, poverty-stricken areas and regions with lower levels of coordinated regional development. In addition, the digital economy exhibits significant positive spatial-spillover effects on air quality in adjacent regions, effectively inhibiting cross-regional diffusion of atmospheric pollution. Mechanism analysis further indicates that the digital economy achieves air-quality improvement in border regions primarily through breaking down regional market segmentation and enhancing environmental tracking and regulatory capacity. This study’s findings help address the challenge faced by developing countries during industrial transformation, namely, that the border regions tend to become “pollution havens.” The findings contribute to improving overall environmental-governance effectiveness.

## Introduction

China is among the world’s largest energy producers and consumers as well as among the countries with the most severe air pollution [1]. To improve air quality, the Chinese government has introduced multiple governance measures and achieved preliminary results [2]. During the current process of industrial structural transformation and upgrading, strict environmental-pollution controls have been implemented to increase enterprise-pollution costs, compelling polluting enterprises to transform or relocate. However, due to long-standing market segmentation and cross-border regulatory-tracking challenges, border regions have become the preferred destinations for polluting enterprises’ relocation [3]. Meanwhile, local governments often tend to adopt beggar-thy-neighbor strategies when addressing environmental-pollution issues, deliberately relaxing environmental regulation in border regions [4] and making them safe havens for polluting enterprises, thereby leading to pollution indicators significantly higher than those in non-border regions [5]. To address air-pollution problems in border regions, the Chinese government has implemented institutional arrangements such as the Air Pollution Prevention and Control Action Plan, the Blue Sky Protection Campaign, and the Central Environmental Protection Inspection, as well as regional air-pollution joint prevention and control mechanisms. Extensive literature has theoretically and empirically demonstrated how these policies have improved air quality in border regions [6].

However, existing governance measures often play only a short-term positive role, and border regions continue to experience significant air-pollution problems [7]. The fundamental reason lies in the institutional tension between centralized environmental policies and decentralized local administrative-management systems, which results in insufficient intrinsic incentives for local governments to engage in border-pollution governance. China’s Ministry of Ecology and Environment has repeatedly noted in reports that some local governments still tolerate and protect polluting enterprises; even after central environmental-protection inspections identify problems, phenomena such as false rectification and perfunctory responses persist, leading to frequent pollution rebounds. This is particularly evident in atmospheric-pollution governance, which is characterized by its cross-regional scope, strong diffusion, and high difficulty in monitoring, and where governance effectiveness remains unsatisfactory. Due to the obvious spatial-spillover nature of atmospheric pollution, cross-border collaborative in governance has become key to enhancing its effectiveness [8]. However, China currently lacks effective oversight mechanisms and clearly defined accountability frameworks across regions, in which limits the outcomes of efforts at collaborative governance. To enhance the efficiency of transboundary air pollution control, China must explore new approaches that incentivize local governments’ intrinsic motivation.

The rise of the digital economy (DE) provides new insights for atmospheric-pollution governance in border regions. According to the “G20 Digital Economy Development and Cooperation Initiative” released at the G20 Hangzhou Summit, a DE is defined as a broad range of economic activities that use digitized knowledge and information as the key factor in production, modern information networks as important carriers, and information and communication technology as important drivers for efficiency improvement and economic-structure optimization. This new economic approach can improve air quality in border regions by promoting the construction of unified markets and reducing cross-border regulatory challenges. A DE can construct an efficient and open integrated market system by precisely matching cross-regional supply and demand, improving the visibility of trading parties, and weakening information asymmetry [9]. The establishment of a unified market helps internalize negative pollution externalities, thereby reducing local governments’ self-interested motives for “beggar-thy-neighbor” strategies.

Meanwhile, breakthrough progress in next-generation digital information technologies represented by big data, artificial intelligence (AI), cloud computing, and the Internet of Things (IoT) provides strong technical support for atmospheric-pollution governance in border regions. The application of emerging digital technologies not only enhances regulatory departments’ technical capacity to track atmospheric-pollution emission sources [10], further clarifying responsibility boundaries for the emissions, but it also provides more convenient tools and channels for public participation in environmental supervision, enabling timely access to pollution information and effective feedback [11]. Enhanced collaborative oversight capacity between government and the public can effectively strengthen constraints on pollutant emissions in border regions, with this effect being particularly pronounced in developing countries [12].

Based on panel data from Chinese inter-provincial border cities during the period 2014–2023, this study systematically evaluates the impact of digital-economy development (DED) on air quality in border regions by constructing DE-measurement indicators. Empirical results show that DED significantly improves air quality in border regions, and the core conclusions hold following a series of robustness tests and endogeneity controls. Heterogeneity tests reveal that the positive impact of DED on border-region air quality is more prominent in non-contiguous, poverty-stricken areas and regions with lower levels of regional coordinated development. In addition, a DE has significant positive spatial-spillover effects on adjacent regions, effectively inhibiting cross-regional atmospheric-pollution diffusion. Further mechanism tests confirm that DED affects border-region air quality by promoting market unification and enhancing tracking and regulatory capacity.

This study’s marginal contributions are mainly reflected in three respects. First, it provides a novel analytical perspective. Existing literature predominantly focuses on centralized environmental policies and administrative regulations, emphasizing how external constraints affect pollution governance. We diverge from this approach by applying digital economy theory to border-region air pollution. This framework reveals how digital infrastructure can strengthen intrinsic governance incentives among local governments, moving beyond a reliance solely on top-down enforcement.

Second, we address a critical research gap in spatial focus. Previous studies of regional environmental issues typically treat spatial units as homogeneous, neglecting the unique governance challenges in border areas created by administrative boundaries. Free-riding behavior among neighboring jurisdictions transforms these regions into regulatory blind spots and pollution havens, exacerbating spatial heterogeneity in air quality and creating governance imbalances. Resolving pollution challenges in border areas is therefore essential for improving the overall effectiveness of environmental governance. This study examines inter-provincial border cities to address this overlooked dimension of regional pollution control.

Third, we identify dual transmission mechanisms. Through systematic analysis of air pollution drivers in border regions, we demonstrate how the digital economy influences emissions through two pathways: market integration and enhanced regulatory capacity. This mechanistic framework advances our theoretical understanding of the digital economy’s environmental governance role while providing an evidence-based foundation for targeted policy interventions.

The remainder of this paper is arranged as follows: Section 2 provides theoretical analysis of the mechanisms through which a DE affects air quality in border regions and proposes corresponding research hypotheses. Section 3 introduces the research design, including data sources, variable descriptions, and model construction. Section 4 conducts an empirical analysis, presents the main regression results, endogeneity and robustness tests, heterogeneity analysis, and spatial-spillover analysis. Section 5 provides further discussion, mainly mechanism analysis, while Section 6 summarizes the main conclusions and proposes the policy implications.

## Mechanism analysis and research hypotheses

Compared with the causes of air pollution within provincial boundaries, those of air pollution in border regions are more complex and possess distinct characteristics, primarily manifested in the two ways. On the one hand, due to the unique geographical location of border areas, market-segmentation phenomena are prevalent, hindering the effective internalization of pollution externalities and resulting in a lack of governance incentives for local governments. Air pollution in border regions possesses, to some extent, the attributes of “public goods”. Border-region governments can obtain economic benefits such as employment and tax revenue from enterprise air pollution; however, due to the mobility of air pollution, these regions only bear part of the cost [13].

Meanwhile, environmental-governance investment in border regions has positive externalities, with governance effects benefiting not only the local area but also diffusing to adjacent regions [14]. Under conditions of limited resources, local governments are more inclined to adopt “free-riding” strategies, namely, reducing governance investment in border areas and allocating more resources to local core regions. Against this backdrop, local governments often lack motivation to actively govern border air pollution and may even attract high-pollution enterprises through relaxed environmental regulations and preferential policies [15]. However, by establishing a unified market system, governments can strengthen economic linkages between adjacent regions, thereby stimulating local governments’ intrinsic motivation to address transboundary pollution [16], which fundamentally contributes to resolving air pollution issues in border areas.

The development of a DE provides strong support for constructing a large, national, unified market. For a long time, China’s system of fiscal decentralization has, to some extent, intensified competition between local governments. Different regional governments, to prevent others from “free-riding,” often establish market barriers to restrict infrastructure sharing and the free flow of factors such as labor and capital, leading to obvious market segmentation between geographically adjacent administrative regions [17]. This segmentation not only limits consumers’ ability to access diversified products across regions but also significantly increases transaction costs for inter-regional enterprise cooperation [18], thereby weakening economic associations between border regions and making it difficult for negative externalities between regions to achieve internalization.

The rise of the DE has broken through this predicament, significantly enhancing economic connections between regions. Unlike traditional production factors, digital elements possess the characteristics of high replicability, strong mobility, and a non-consumptive nature, effectively penetrating temporal and spatial barriers. The rapid development of emerging digital technologies such as cloud computing, big data, IoT, mobile internet, and AI has significantly improved information-transmission efficiency and complex data-processing capabilities, providing a solid technological foundation for inter-regional information sharing and knowledge diffusion [19]. Meanwhile, the widespread application of digital technology has reduced information-communication costs, enabling consumers to conveniently access products and services from other regions through digital platforms; this promotes inter-enterprise cross-regional collaboration and thus expands and deepens economic connections between regions [20]. Therefore, the development of the DE fundamentally weakens market barriers between administrative divisions and accelerates the formation of a unified large market [21]. As regional economic linkages strengthen, negative externalities generated in border regions become easier to internalize, thereby helping to enhance local governments’ enthusiasm and sense of responsibility in environmental governance, especially in border air-pollution prevention and control.

Meanwhile, border regions face typical “prisoner’s dilemma” game situations in air-pollution governance, where local governments tend to adopt governance strategies that deviate from socially optimal outcomes. Constrained by the artificial division of administrative boundaries, high information asymmetry arises between border parties regarding waste-gas emissions and governance behaviors, and regulatory constraint mechanisms are relatively weak. When local governments decide whether to undertake pollution governance, they must consider not only local interests but also the action strategies of neighboring regions [22]. If adjacent regions adopt pollution-governance strategies, the optimal choice for local governments is a “free-riding” strategy, as this allows them to obtain employment and tax-revenue increases from environmental pollution while avoiding governance costs. Furthermore, even if neighboring regions choose pollution strategies, from the perspective of maximizing short-term economic interests for local governments, their optimal response strategy may still be to tolerate or acquiesce in polluting behaviors in order to remain attractive to external investment. This non-cooperative game leads to a “race to the bottom,” where governments compete to relax environmental standards in pursuit of relative competitive advantages, ultimately resulting in a systematic decline in the effectiveness of environmental governance [23]. Therefore, to maximize self-interest, regardless of whether the other party chooses pollution strategies, the optimal strategy for local governments is to choose to tolerate pollution emissions, which allows them to develop the economy while not having to fully bear the consequences.

However, such behavior may generate spatial spillover effects, whereby pollutants migrate to border regions and create pollution havens [24]. This is particularly problematic for air pollution, which exhibits high atmospheric mobility and cross-boundary dispersion. Consequently, without collaborative governance mechanisms, pollution control costs escalate across neighboring jurisdictions, reinforcing incentives for non-cooperative strategies and free-riding behavior. In addition, border regions are typically far from administrative centers, with weak public environmental-perception capabilities; given the concealed and long-term nature of pollution problems, the likelihood of pressure and intense supervision by public opinion is limited, significantly reducing the probability of government officials being held accountable for environmental negligence [25]. Therefore, the key to resolving border pollution-governance dilemmas lies in strengthening environmental-supervision capabilities and clarifying how responsibility for border air pollution is attributed, thereby compelling local governments to become more willing to implement air-pollution governance and transform their approach from “passive response” to “active cooperation.”

The development of the DE not only enhances the ability to precisely track air-pollution sources through digital technology but also leverages information platforms to expand the breadth and depth of public participation in environmental governance; this helps to strengthen those mechanisms that constrain pollution-emission behaviors in border regions. First, the DE provides important technological support for local governments to track air-pollution sources in border regions. Such regions are typically far from administrative centers, and this geographical distribution leads to an increasingly prominent “geographical decay effect” in local governments’ environmental regulation of border regions—that is, the farther the distance, the weaker the regulatory intensity, allowing polluting enterprises to exploit spatial distance to evade environmental constraints and increase pollutant emissions [26]. Moreover, constrained by the limitations imposed by administrative boundaries, local governments often cannot implement cross-border regulation, exacerbating regulatory-failure problems. With the development of the DE, environmental-governance methods based on cutting-edge technologies such as big data and satellite remote sensing continue to emerge, greatly enhancing government capabilities in air-pollution monitoring, early warning, and tracking. Leveraging emerging technologies such as cloud computing, AI, and virtual reality, governments can not only access pollution-emission data in real-time but can also simulate pollution-diffusion pathways, identifying pollution sources across administrative boundaries and thereby clarifying environmental responsibility and reducing transaction costs for inter-local coordinated governance.

Second, the DE expands channels for public participation in environmental supervision through information platforms, enhancing the transparency and constraint power of social governance. As a new economic form operating on modern information and communication networks, the DE significantly reduces information-dissemination thresholds and time delays [27]. The public can quickly access regional air-pollution conditions, enterprise-emission data, and policy-implementation status through network channels, not only deepening public awareness of pollution hazards but also achieving an exponential dissemination of environmental issues through social media, thus increasing exposure rates for pollution incidents. Pressure from public-opinion supervision has gradually become an important external force that compels local governments to fulfill their responsibilities for environmental governance in practice. Particularly for border regions, strong social attention often directly affects local officials’ performance evaluation, thereby enhancing their enthusiasm for pollution governance.

Based on the above analysis, we proposes the following research hypotheses:

H1: DED is conducive to improving air quality in border regions.

H2: The DE improves air quality in border regions by breaking market segmentation and enhancing environmental tracking and regulatory capabilities.

## Research design

### Sample Selection and Data Sources

We examined inter-provincial border cities from 2014 to 2023 for two primary reasons. First, data on air quality and the digital economy are difficult to obtain at lower administrative levels (counties and districts), potentially compromising research reliability. Second, inter-provincial border cities serve as critical nodes for regional economic interaction, making their environment-economy nexus particularly valuable for study. Our sample comprised 188 cities, representing 66.43% of all prefecture-level cities in China. Using the 2024 edition of China’s provincial administrative boundary vector map, we identified border cities distributed across eastern, central, and western economic regions. This sampling strategy ensured adequate spatial representation and statistical robustness.

Four main data sources were used for the sample cities. (1) Spatial boundary identification: Based on the 2024 version of China’s administrative-division vector map, we used the ArcGIS software to extract urban-boundary information, identify cities in adjacent provinces, and use them as samples for analysis. (2) Air Quality Index (AQI): We used official data released by the Ministry of Ecology and Environment for measurement, with higher AQI values indicating more severe air pollution. (3) Socioeconomic and climate data: We sourced levels of economic development, population, and social statistics for each city from the China City Statistical Yearbook, while natural-climate variables (such as temperature, precipitation, and wind speed) were derived from the “China Ground Climate Data Daily Value Dataset V3.0.” (4) Data cleaning and completion: Cities with excessive missing data were excluded, and linear interpolation was used to address minor data gaps in remaining cities, ultimately generating 1,880 city-year sample observations.

### Model construction and variable description

To verify the impact of DED on air quality in border regions, we constructed the following benchmark regression model:


$$\begin{array}{c} {AQI}_{i,t+\text{1}}=\alpha_{\text{0}}+\alpha_{\text{1}}{DE}_{i,t}+\alpha_{\text{2}}X_{i,t}+{\mu_{i}+\varepsilon }_{i,t} \end{array}$$ (1)

where i represents border cities and t represents years, and the dependent variable, ${AQI}_{i,t+1}$, is the AQI of city i in year t + 1. The core explanatory variable, ${DE}_{i,t}$, is the DED level of city i in year t; Controls, $X_{i,t}$, represent a set of control variables; $\mu_{i}$ is the city-fixed effect, used to capture time-invariant characteristics at the city level; $\varepsilon_{i,t}$ is the random error term. The model analysis focuses on coefficient $\alpha_{1}$. If the estimation results for $\alpha_{1}$ is significant and negative, it indicates that border regions with better DED show more pronounced air-quality improvement.

The dependent, independent, and control variables were as follows

### Dependent variable

We used the AQI as the measurement indicator for the dependent variable. The data were obtained from materials published by China’s Ministry of Ecology and Environment, and the index is the most authoritative evaluation indicator for air-quality in China, covering concentrations of air pollutants such as SO₂, NO₂, PM₁₀, PM₂.₅, and CO and comprehensively reflecting local air-pollution conditions. Given the possibility of a potential bidirectional causal relationship between the DE and air quality—that is, rather than DED improving air quality, regions with good air quality may be more likely to attract digital-industry agglomeration and development—we selected the AQI of the following year as the dependent variable to avoid potential endogeneity bias. In addition, considering that PM₂.₅ is the main pollutant forming haze and poses significant harm to human health, as well as being a widely studied indicator in atmospheric environmental research, we used the annual average PM_2.5_ values of cities to replace AQI in robustness analysis to test the robustness of model conclusions.

### Independent variable

The core explanatory variable in this study is the DED level. As the third major economic form after agriculture and industry, the DE has not yet been separately listed in traditional national economic classifications; thus, comprehensive indicators must be constructed to quantify its development level. Previous domestic research typically relies on two aspects—internet development and digital inclusive finance—to assess urban DE; however, this method has limited coverage and cannot fully capture the essence of a DE [29]. The G20 Hangzhou Summit Digital Economy Development and Cooperation Initiative’s definition of a DE provides reference for constructing indicators in this study: a DE should encompass three levels: “digital infrastructure,” “digital industrialization,” and “industrial digitalization.” Based on the this framework, we designed the following indicator system to measure DED level: (1) digital infrastructure, including the number of DE enterprises, registered capital, and the number of internet broadband access users and mobile-phone users per 100 people; (2) digital industrialization, measured by the proportion of computer services and software practitioners among urban-unit employees and per-capita telecommunications-business volume; and (3) industrial digitalization, reflected by the digital inclusive finance index (calculated using the construction method proposed by Feng et al. [29]), and the digital government development index. Digital government data are directly adopted from the China Government Website Performance Evaluation Report (2011–2016) and Government Electronic Service Capability Index Report (2017–2023) published by Nanjing University.

Following Feng et al. [28], we employed the entropy weight method to construct a comprehensive digital economy development index at the prefecture-level city scale. This indicator effectively mitigates potential biases arising from subjective weight assignment in previous studies and, when integrated with the aforementioned definition, enables a more comprehensive and accurate measurement of digital economy development levels. To ensure the robustness of our findings, we further validated our results in robustness checks by measuring digital economy development from two alternative perspectives: internet development and digital inclusive finance.

### Control variables

To accurately identify the impact of DED on air quality in border regions, we simultaneously controlled for economic, social, and natural environmental factors that might interfere with air quality in the regression model: (1) Economic and social factors included regional per-capita gross domestic product (pgdp), secondary industry proportion, residential consumption, and road infrastructure; (2) Natural factors included temperature, precipitation, and wind speed.

The descriptive statistics for the main variables are shown in Table 1. Using the AQI as an example, the minimum and maximum values among sample cities are 3.51 and 5.16, respectively, reflecting significant differences in air quality among border cities. Meanwhile, DED levels also show high variability: the highest comprehensive DE index is 0.686, while the lowest is only 0.032. Such disparities indicate severe imbalances in DED across different regions, and the resulting “digital divide” may weaken the scale effects of the DE.

**Table 1:** Descriptive statistics of variables.

Variable Name	Variable Symbol	Mean	Std. Dev.	Min	Max
Air Quality Index	AQI	4.323	0.260	3.531	5.167
Digital Economic Development Level	DE	0.128	0.068	0.032	0.686
Per-Capita GDP	pgdp	3.912	0.495	2.320	5.579
Secondary-Industry Proportion	sec	0.426	0.102	0.116	0.755
Road-Infrastructure Density	road	1.136	0.553	0.025	5.881
Residential Consumption Level	consume	4.209	0.877	1.718	6.865
Precipitation	rain	6.880	0.553	3.822	8.033
Wind Speed	wind	8.802	1.534	5.142	14.194
Temperature	tem	14.647	4.771	−0.760	25.715

## Empirical analysis

### Baseline regression results

The baseline regression results are summarized in Table 2. Column (1) includes only the core explanatory variables. The results show that the DE coefficient is negative and highly significant at the 1% level, providing preliminary validation of the hypothesis that DED helps improve air quality in border regions. Columns (2) and (3) sequentially add economic-social and natural-environment control variables to the specification in column (1). The DE coefficient remains significant and negative, indicating that the improvement effect of DED on air quality in border regions holds when socioeconomic and natural conditions are controlled for.

**Table 2:** Baseline regression results.

Variable	(1)	(2)	(3)
DE	−1.107^***^	−0.379^***^	−0.314^***^
	(0.069)	(0.059)	(0.059)
pgdp		−0.276^***^	−0.248^***^
		(0.014)	(0.015)
sec		1.053^***^	0.996^***^
		(0.059)	(0.046)
road		−0.014	−0.010
		(0.012)	(0.012)
consume		0.094^***^	0.101^***^
		(0.011)	(0.011)
rain			0.010
			(0.012)
wind			−0.004
			(0.005)
tem			−0.055^***^
			(0.008)
Constant term	Yes	Yes	Yes
Observations	1692	1692	1692
R^2^	0.147	0.511	0.529

Note: Standard errors in parentheses are robust standard errors adjusted for clustering at the city level. ***, **, and * denote statistical significance at the 1%, 5%, and 10% levels, respectively. This notation applies throughout.

### Endogeneity analysis

A bidirectional causal relationship may exist between the DE and air quality in border regions. This means that rather than DED improving air quality in border regions, areas with good air quality may be better able to attract digital industries, thereby enhancing local DE levels. To further mitigate the endogeneity issues that may arise from this situation, we drew on the shift-share method [29] to construct instrumental variables (IVs) and used two-stage least squares (2SLS) for estimation. Specifically, the product of the number of fixed telephones per 100 people in cities in 1984 and the annual national information-technology service revenue were used as the IV for the DE. We selected the number of fixed telephones per 100 people in cities in 1984 as the share component for two reasons: First, historical telecommunications infrastructure affects subsequent internet-technology development, thereby influencing local DE levels and satisfying the requirements for IVs. Second, given that telecommunications infrastructure construction exerts relatively minimal impact on atmospheric environmental quality, particularly over extended temporal intervals [7], this study posits that the number of fixed telephone lines per 100 persons in 1984 is exogenous to air quality in border regions during 2014–2023, thereby satisfying the exogeneity requirement for instrumental variables.

It should be noted that the number of fixed telephones per 100 people in cities in 1984, as cross-sectional data, cannot be directly used for panel-data econometric analysis; thus, a time-varying variable is required to construct panel IVs. Therefore, we introduced annual national information-technology service revenue as the shift component and multiplied it by the number of fixed telephones per 100 people in cities in 1984 (share component); we then applied logarithmic transformation to the result to reduce the errors caused by dimensional issues [28].

Column (1) of Table 3 presents the first-stage regression results, where the IV coefficient is highly significant at the 1% level, indicating that it satisfies the relevance assumption. Column (2) of Table 3 shows the second-stage regression results, with the DE coefficient being significant and negative. This indicates that even after using IVs to eliminate endogeneity, the DE still has a significant positive effect on air-quality improvement in border regions. Furthermore, various validity tests support the conclusions of this study: the LM statistic P-value in the under-identification test is 0, significantly rejecting the null hypothesis of “insufficient IV identification”; and the F-statistic in the weak-IV test is greater than the critical value at the 10% level of the Stock-Yogo test, indicating that no weak-IV problem exists.

**Table 3:** Endogeneity analysis.

Variable	2SLS		DID
First Stage	Second Stage
(1)	(2)	(3)
iv	0.012^***^		
	(0.002)		
DE		−2.927^***^	
		(0.712)	
wide_DID			−0.391^***^
			(0.069)
Kleibergen-Paap Wald rk F	67.776		
Kleibergen-Paap rk LM	45.589 (0.000)		
Control Variables	Yes	Yes	Yes
Observations	1692	1692	1692
R^2^	0.480	0.218	0.387

Given that the selection of any instrumental variable inevitably involves a degree of subjectivity, which may introduce bias into regression results, we took additional steps to further mitigate potential endogeneity concerns, using a difference-in-differences (DID) regression based on the “Broadband China” pilot policy. “Broadband China” is a digital-infrastructure development strategy launched by the Chinese government in 2013, which selected 120 cities as pilots in three rounds during 2014, 2015, and 2016, focusing on enhancing broadband-user scale, network speed, and coverage. After the construction period (approximately three years), the pilot cities’ broadband-access capabilities and penetration rates are expected to rank among the top nationally, thereby promoting DED. Following Feng et al. [28], we used the “Broadband China” pilot as an exogenous policy shock to establish the following DID model:


$$\begin{array}{c} {AQI}_{i,t+1}=\beta_{\text{0}}+\beta_{\text{1}}wide_{{DID}_{i,t}}+\beta_{\text{2}}X_{i,t}+{\mu_{i}+\varepsilon }_{i,t}, \end{array}$$ (2)

where ${AQI}_{i,t+1}$ represents the air-quality level of city i in year t + 1, while $wide\_{DID}_{i,t}$_ is a dummy variable that equals 1 from year t onward if city i becomes a “Broadband China” pilot in year t and 0 otherwise (the control group always equals 0). The regression results are shown in column (3) of Table 3, where the estimated coefficient of $wide\_{DID}_{i,t}$ is significant and negative, indicating that the “Broadband China” pilot effectively improved air quality in pilot cities. This demonstrates that after using the DID method to eliminate endogeneity problems, the finding that DED improves air quality in border regions remains significant.

## Robustness tests

### Replacing the dependent variable

PM_2.5_ is among the core indicators for assessing air quality and was the primary pollutant in the “Beijing Haze Event” of 2013. Considering the authority and accessibility of data sources, we adopted PM_2.5_ grid data converted from Aerosol Optical Depth (AOD) by the National Aeronautics and Space Administration (NASA) based on multiple instruments, including MODIS, MISR, and SeaWiFS. Using ArcGIS, these grid data were aggregated to the border-city level, and their natural logarithm was used as a proxy indicator for air quality in the regression. The regression results are shown in column (1) of Table 4: the DE coefficient remains significant and negative, further validating the improving effect of DE on air quality in border regions. In addition, considering that annual averages may underestimate the governance effect of DE during heavily polluted periods, we further used “the proportion of days with good air quality within the year” as an alternative dependent variable for regression. The results are shown in column (2) of Table 4: the coefficient of the core explanatory variable remains significant and positive, indicating that DED significantly increases the proportion of days with good air quality in border‌‌ regions.

**Table 4:** Robustness tests.

Variable	Replacing the Dependent Variable		Replacing the Core Explanatory Variable	Removing Outliers	Removing Special Observations
**(1)**	**(2)**	**(3)**	**(4)**	**(5)**
DE	−0.329^***^ (0.075)	0.242^***^ (0.064)	−0.830^***^ (0.077)	−0.314^***^ (0.059	−0.284^***^ (0.059)
Control Variables	Yes	Yes	Yes	Yes	Yes
Observations	1880	1880	1692	1692	1611
R^2^	0.767	0.387	0.555	0.529	0.523

### Replacing the core explanatory variable

Following Tao et al. [30] regarding DE measurement, we measured the DED level using two aspects for robustness testing: internet development and digital inclusive finance. The regression results are shown in column (3) of Table 4: after changing the measurement method for the core explanatory variable, the DE coefficient remains significant and negative, and the results still hold.

### Removing the influence of outliers

The presence of outliers in a sample can distort regression estimation results, causing coefficients to deviate from their true levels. To mitigate this influence, we conducted a 1% sample winsorization, truncating the most extreme data at the 1% percentile and replacing them with boundary values. This method can reduce the impact of extreme values on regression results while preserving sample size. The regression results after winsorization are shown in column [4] of Table 4: the DE coefficient remains significant and negative, indicating that after removing the interference of extreme values, the improving effect of DE on air quality in border regions still holds.

### Removing special observations

Cities located on national borders may be affected not only by domestic factors but also by more complex international factors (such as cross-border pollution, economic differences, and policy spillovers). To ensure that model estimation is not biased due to these potential cross-border influences, we further removed all cities located on national borders from the sample and re-estimated the regression model. The regression results after removing special observations are shown in column [5] of Table 4: the DE coefficient variable remains significant and negative, indicating that after excluding the influence of border cities, the improving effect of DE on air quality in border regions remains significant. It is noteworthy that the regression results are not statistically significant due to the limited sample size of only nine border cities examined in this study (detailed regression results are presented in S1 Table). Further research on this topic warrants deeper investigation by future scholars.

### Heterogeneity analysis

Due to the barriers of geographical and administrative boundaries, the economic-development level of border regions is relatively lagging, and the concentration of polluting enterprises may hinder their sustainable development. Therefore, we divided the sample into two groups based on the Chinese government’s 2012 lists “Contiguous Poverty-Stricken Areas” and “Non-Contiguous Poverty-Stricken Areas”. We then re-estimated the regression model to test the applicability of the improving effect of DE on air quality in border regions across different types of areas.

The regression results in columns (1) and (2) of Table 5 show that DE significantly improves air quality in non-contiguous, poverty-stricken areas, while this effect is not significant in contiguous, poverty-stricken ones. This is because digital-infrastructure construction in contiguous, poverty-stricken areas is backward, and the DED level is extremely limited, while the effect of DE on environmental improvement often has a threshold effect [31], which prevents the positive impact of digital economy development on air quality in border regions from being fully realized.

**Table 5:** Heterogeneity analysis.

Variable	Contiguous Poverty-Stricken Areas		Regional Coordinated Development Level	
	Yes	No	Yes	No
	**(1)**	**(2)**	**(3)**	**(4)**
DE	−0.099 (0.143)	−0.334^***^ (0.063)	−0.213^**^ (0.099)	−0.308^***^ (0.072)
Inter-group Coefficient Differences	Contiguous vs. Non-contiguous Poverty Areas		Low vs. High Coordination Development Level	
	0.235 [0.000]		−0.094 [0.029]	
Control Variables	Yes	Yes	Yes	Yes
Observations	468	1224	432	1260
R^2^	0.551	0.528	0.608	0.509

Breaking through inter-provincial boundary restrictions and achieving cross-provincial coordination is an important approach for China to build a unified national market. Based on the 19 urban-agglomeration plans proposed by the Chinese government in the “14th Five-Year Plan,” we classified the relatively mature Yangtze River Delta, Pearl River Delta, Beijing-Tianjin-Hebei, Middle Yangtze River, and Chengdu-Chongqing urban agglomerations as the high-coordination group, while other regions were classified as the low-coordination group for regression analysis. The results shown in columns (3) and (4) of Table 5 indicate that although DED can significantly improve air quality in border regions in both sample groups, the coefficient for the low-coordination group is significantly larger, suggesting that DE has a more significant effect on air-quality improvement in regions with lagging coordinated development. This is because in low coordination regions, due to loose economic ties between provinces, market-boundary effects are more pronounced and air-pollution problems are more severe. DED precisely reduces boundary environmental pollution by breaking down market segmentation [32].

### Spatial-spillover effects analysis

On the one hand, as air pollution is extremely prone to dispersion, border air quality is the result of multi-party gaming among adjacent provinces. Therefore, when exploring border air-pollution issues, attention should also be paid to the influence of multiple factors such as the economic structure, industrial production levels, and natural environment of neighboring regions. The regression results shown in column (1) of Table 6 indicate that even after controlling for the influence of neighboring regions’ economic, social, and natural conditions, the impact of DE on air quality in border regions remains significant and negative at the 1% level.

**Table 6:** Spillover effects analysis.

Variable	Spatial Spillover Effects	Total Effect	Direct Effect	Indirect Effect
	**(1)**	**(2)**	**(3)**	**(4)**
DE	−2.106^***^ (0.208)	−1.726^***^ (0.478)	−0.007^***^ (0.056)	−1.718^***^ (0.471)
Control Variables	Yes	Yes	Yes	Yes
Observations	1880	1880	1880	1880
R^2^	0.397	0.397	0.397	0.397

On the other hand, DED can break traditional market segmentation, enhance inter-regional connections at the socioeconomic level, and promote cross-border transmission of green knowledge and technology. Furthermore, the improvement of digital technology can enhance local governments’ capabilities in monitoring, tracing, and regulating pollution sources, technically suppressing the gaming behavior of atmospheric emissions in border regions and forcing adjacent regions to address air pollution. Therefore, DE can not only improve local air conditions but also produce significant environmental impacts on neighboring regions through market and technological spillovers. This viewpoint has been supported by extensive empirical analysis. For example, Arogundade and Hassan [33] used a spatial Durbin model (SDM) and found that DED was positively correlated with air quality and had obvious spatial-spillover effects. To test whether the improving effect of DED on air quality in border regions has spatial-spillover effects, we constructed a General Nested Spatial (GNS) econometric model, as specified below:


$$\begin{array}{c} {AQI}_{i,t}=\gamma_{\text{0}}+\gamma_{\text{1}}{\text{W}}_{ij}{AQI}_{i,t}+\sum \gamma_{\text{2}}X_{i,t}+\theta {\text{W}}_{ij}\sum \gamma_{\text{2}}X_{i,t}+{\mu_{i}+v}_{i,t} \end{array}$$ (3)

$$\begin{array}{c} \nu_{it}=\lambda W_{ij}\nu_{i,t}+u_{i,t} \end{array}$$ (4)

where $W_{ij}$ represents the spatial weight matrix between regions i and j, constructed based on the geographical distance between city administrative centers; $\gamma_{\text{1}}$ is the spatial autoregressive coefficient, measuring the transmission effect of neighborhood air quality; θ is the coefficient of explanatory variables on the spatial lag term, reflecting spatial-spillover effects; $\mu_{i}$ controls for regional fixed effects; λ represents the spatial-error autocorrelation coefficient, reflecting the spatial clustering of error terms; and $X_{i,t}$ represents the set of core explanatory and control variables.

Following Elhorst [34], we adopted both “specific-to-general” and “general-to-specific” methods to compare and select spatial econometric models. Through this bidirectional testing, we found that the SDM performed better in LR and Hausman tests and therefore selected it as the optimal model, specified as follows:


$$\begin{array}{c} {AQI}_{i,t}=\gamma_{\text{0}}+\gamma_{\text{1}}{\text{W}}_{ij}{AQI}_{i,t}+\omega_{\text{1}}\text{D}{\text{E}}_{i,t}+\sum \gamma_{\text{2}}X_{i,t}+W_{ij}\left(\omega_{\text{1}}\text{D}{\text{EI}}_{i,t}+\sum \gamma_{\text{2}}X_{i,t}\right)+{\mu_{i}+v}_{i,t} \end{array}$$ (5)

To test whether neighboring cities are also affected by local DED, we examine the results shown in column (1) of Table 6: the DE variable has a significant and negative coefficient at the 1% level. This indicates that DED in border regions has positive spatial-spillover effects on air quality, which means that local DED significantly improves air quality in surrounding cities.

We also applied partial differential analysis to the SDM, decomposing the total effect into direct and indirect effects. This effect-decomposition capability is an important feature that distinguishes spatial regression models from traditional non-spatial models and can reflect the differential impacts of neighboring regional variable changes on local and surrounding areas [35]. The specific results are shown in columns (2), (3), and (4) of Table 6. Evidently, the absolute value of the indirect effect is greater than that of the direct effect, which means that DED does improve air quality in surrounding areas through spatial-spillover mechanisms.

## Mechanism testing

### Breaking regional market-segmentation mechanism

Under traditional economic development models, market fragmentation in border regions impedes the internalization of pollution’s negative externalities, perpetuating long-standing “beggar-thy-neighbor” pollution emission practices. The rise of the digital economy reduces cross-regional cooperation costs through efficient information transmission, breaking down administrative barriers and fostering collaborative win-win outcomes among adjacent economies. When the negative externalities of pollution emissions harm local interests, local governments’ incentives to exploit border pollution for economic development are significantly diminished, thereby contributing to improved air quality in border areas. To test this mechanism, we adopted the relative price variance method following Lv and He [36] to calculate the market segmentation index, assessing the degree of market fragmentation across different regions. Specifically, by computing the variance of relative price fluctuations across seven categories of consumer price indices, we can capture the magnitude of internal market barriers. A higher market segmentation index indicates more severe impediments to resource and information flows, with market efficiency and competitiveness being correspondingly constrained.

In addition to employing the relative price variance method to calculate the market segmentation index, we also drew on Sheng and Mao [37] to measure market integration using the price index approach as an alternative indicator of market fragmentation, where higher market integration corresponds to lower market segmentation. The results demonstrate that the digital economy generates more pronounced air quality improvements in border regions characterized by higher market integration, thereby strengthening the robustness of our mechanism validation. Detailed results are presented in S2 Table.

To exclude the influence of digital economy development on market segmentation, we divided the sample into high and low groups based on the median market segmentation index in 2013 and conducted separate regression analyses. As shown in columns (1)-(2) of Table 7, the coefficient of the core explanatory variable is significantly negative at the 10% level in the low market segmentation group, while the coefficient in the high market segmentation group is statistically insignificant. Coefficient difference tests reveal significant disparities between the two groups. Digital economy development exhibits a Matthew effect [38], more substantially strengthening economic linkages in border regions with lower market segmentation, thereby more effectively promoting green growth and environmental improvement.

**Table 7:** Mechanism testing.

Variable	Low Market-Segmentation Level	High Market-Segmentation Level	Weak Environmental-Regulation Level in Adjacent Areas	Strong Environmental-Regulation Level in Adjacent Areas
	**(1)**	**(2)**	**(3)**	**(4)**
DE	−0.162^*^ (0.092)	0.101 (0.098)	0.108 (0.096)	−0.198^**^ (0.092)
Inter-group Coefficient Differences	Low vs. High Market Segmentation		Weak vs. Strong Environmental Regulation	
	−0.263 [0.060]		0.307 [0.030]	
Control Variables	YES	YES	YES	YES
Observations	864	828	747	945
R^2^	0.685	0.689	0.698	0.670

### Enhancing environmental tracking and regulatory-capacity mechanism

In cross-jurisdictional environmental governance contexts, both sides of adjacent administrative boundaries often fall into a “prisoner’s dilemma” due to strategic gaming over pollution emissions and abatement investments: when environmental regulatory efficacy is inadequate, the absence of effective constraint mechanisms facilitates collectively irrational pollution discharge behaviors. The intervention of the digital economy reconstructs the environmental governance logic in border regions: on the one hand, digital platforms lower participation barriers for public environmental monitoring, generating social co-governance pressure; on the other hand, technologies such as the Internet of Things (IoT) and big data enable precise pollution source tracing and dynamic monitoring, significantly enhancing regulatory effectiveness. This technology-enabled governance model effectively curtails strategic pollution behaviors in border areas, providing a new paradigm for resolving challenges to cross-regional environmental governance. To empirically test this mechanism, the external environmental governance constraints faced by border regions, namely the intensity of environmental administrative regulation in surrounding areas, needs to be measured. Following Lihui and Chuanqing [39], we used Python to crawl the frequency of 15 environmental protection-related keywords (such as “emission reduction,” “air,” and “haze”) from local government annual work reports to construct an indicator of local environmental-regulation enforcement intensity. Generally, the higher this indicator, the stronger the local government’s environmental regulatory capacity.

To control for the potential feedback effect of digital economy development on the classification of environmental regulation intensity, we used the median value of the environmental regulation enforcement intensity indicator in 2013 as the threshold to divide the sample into “high regulation” and “low regulation” groups, conducting separate regression analyses for each subsample. Simultaneously, to avoid reverse causality concerns, we continued to employ the one-period lagged air quality index as the dependent variable in the mechanism tests. The results are shown in columns (3) and (4) of Table 7: the coefficient for the group with strong environmental regulation in adjacent areas is significantly negative, while the coefficient for the other group is statistically insignificant, with inter-group coefficient difference tests confirming significant heterogeneity between the two subsamples. This indicates that in regions with high regulatory levels, digital economy development more substantially improves local air quality, effectively breaking the “strategic deadlock” in border areas. In China, regions with weak environmental regulatory capacity are often economically underdeveloped areas where lagging digital infrastructure and insufficient technological adoption constrain the environmental governance efficacy of the digital economy.

## Conclusions and implications

Based on panel data from inter-provincial border cities from 2014 to 2023, we constructed a DED index from three dimensions: digital-infrastructure construction, digital industrialization, and industrial digitalization. We then systematically examined the impact of the index on air quality in border regions. We found the following: (1) DED can effectively improve air quality in border regions, and the main conclusions hold after robustness and endogeneity tests; (2) Heterogeneity analysis reveals that the improving effect of the DE on air quality in border regions is more significant in non-contiguous, poverty-stricken areas and those with low coordinated-development levels; (3) Spatial-spillover effect analysis shows that DED improves air quality in surrounding areas through spatial-spillover mechanisms; however, its improvement effect on local air quality is more pronounced; and (4) Mechanism analysis finds that DE can improve air quality in border regions by breaking down regional market segmentation and enhancing environmental tracking and regulatory capacity. Based on these conclusions, we propose the following policy recommendations.

First, develop a digital technology-enabled framework for cross-border pollution governance. Deploy integrated monitoring networks using IoT sensors, remote sensing, and big data analytics to enable precise pollutant source identification and accountability tracking. Simultaneously, advance the intelligent transformation of environmental protection equipment by establishing cross-regional platforms for production factor mobility that leverage energy consumption data sharing and intelligent scheduling algorithms to reduce industrial emissions. These platforms should be complemented with green technology marketplaces facilitating clean production adoption. Finally, create environmental information disclosure portals and citizen engagement tools (e.g., crowdsourced pollution reporting applications) to establish multi-stakeholder governance mechanisms involving government, industry, and civil society across the entire pollution control chain.

Second, strengthen institutional mechanisms for cross-regional digital economy cooperation. Promote shared infrastructure development, including 5G networks and cloud computing centers, to reduce data transmission costs while establishing integrated platforms for environmental monitoring and industrial planning coordination. Develop explicit cooperation frameworks that specify data sharing protocols and benefit distribution mechanisms. Incorporate cooperation performance metrics into local government evaluations to discourage protectionist policies. Create dedicated digital economy investment funds to facilitate technology transfer from developed to less-developed regions and support green manufacturing capacity building. Utilize industrial internet platforms to enable coordinated order management and sharing of production capacity, thereby establishing an integrated system spanning research and development, manufacturing, and market access.

Third, explore integrated pathways linking digital poverty alleviation with sustainable development. Implement comprehensive network coverage initiatives to ensure universal 5G and fiber-optic access in impoverished regions, accompanied by training programs in e-commerce operations and digital collaboration skills. Advance “digital agriculture” models that leverage e-commerce platforms to market specialty agricultural products and establish digital production facilities that fulfill orders from developed regions, thereby creating local employment opportunities. Deploy early warning systems for ecological thresholds to guide investment toward green industries such as eco-tourism and renewable energy. Incorporate both poverty reduction outcomes and environmental indicators into performance evaluations, creating a virtuous cycle of digital empowerment,‌‌ income generation, and ecological conservation.

## Supporting information

S1 Table Appendix 1. (DOCX)

S2 Table Appendix 2. (DOCX)

S1 Date Data 1. (XLSX)

S2 Date Data 2. (XLSX)
