# Supplementary material for: Does digital-economy development improve air quality in Border Regions? Empirical evidence from 188 Chinese cities
Source: PLoS One. 2026 May 14;21(5):e0348514. doi: 10.1371/journal.pone.0348514 (PMC13175366; doi:10.1371/journal.pone.0348514)
Supplement: S1 Table — (DOCX) [file pone.0348514.s001.docx]

**S1 Table Appendix1**

| Variable | Domestic Sample | Border Sample |
| --- | --- | --- |
|  | (1) | (2) |
| DE | -0.284^***^  (0.059) | -0.402  (0.345) |
| Control Variables | YES | YES |
| Observations | 1,611 | 81 |
| R² | 0.523 | 0.718 |
