# Supplementary material for: Does digital-economy development improve air quality in Border Regions? Empirical evidence from 188 Chinese cities
Source: PLoS One. 2026 May 14;21(5):e0348514. doi: 10.1371/journal.pone.0348514 (PMC13175366; doi:10.1371/journal.pone.0348514)
Supplement: S2 Table — (DOCX) [file pone.0348514.s002.docx]

**S2 Table Appendix2**

| Variable | Low Market Integration | High Market Integration |
| --- | --- | --- |
|  | (1) | (2) |
| DE | 0.082  (0.096) | -0.187^**^  (0.094) |
| Control Variables | YES | YES |
| Difference Between Groups (Low – High Market Integration) | 0.270  [0.020] | |
| Observations | 990 | 702 |
| R² | 0.685 | 0.691 |
